# The Use of Specialized Pro-Resolving Mediators in Biomaterial-Based Immunomodulation

**DOI:** 10.3390/jfb14040223

**Published:** 2023-04-15

**Authors:** Ana Beatriz Sousa, Judite N. Barbosa

**Affiliations:** 1 i3S—Instituto de Inovação e Investigação em Saúde, Universidade do Porto, Rua Alfredo Allen, 208, 4200-125 Porto, Portugal; 2 INEB—Instituto de Engenharia Biomédica, Rua Alfredo Allen, 208, 4200-125 Porto, Portugal; 3 ICBAS—Instituto de Ciências Biomédicas Abel Salazar, Universidade do Porto, Rua de Jorge Viterbo Ferreira, 228, 4050-313 Porto, Portugal

**Keywords:** acute inflammation, resolution of inflammation, specialized pro-resolution mediators (SPMs), immunomodulatory biomaterials

## Abstract

The implantation of a biomaterial will lead to the immediate onset of an acute inflammatory response, which is of key importance in shaping the quality of the repair process. However, the return to homeostasis is critical to prevent a chronic inflammatory response that may impair the healing process. The resolution of the inflammatory response is now recognized as an active and highly regulated process, being described as specialized immunoresolvents that have a fundamental role in the termination of the acute inflammatory response. These mediators collectively coined as specialized pro-resolving mediators (SPMs) are a family of endogenous molecules that include lipoxins (Lx), resolvins (Rv), protectins (PD), maresins (Mar), Cysteinyl-SPMs (Cys-SPMs) and n-3 docosapentaenoic acid-derived SPMs (n-3 DPA-derived SPMs). SPMs have important anti-inflammatory and pro-resolutive actions such as decreasing the recruitment of polymorphonuclear leukocytes (PMNs), inducing the recruitment of anti-inflammatory macrophages, and increasing macrophage clearance of apoptotic cells through a process known as efferocytosis. Over the last years, the trend in biomaterials research has shifted towards the engineering of materials that are able to modulate the inflammatory response and thus stimulate appropriate immune responses, the so-called immunomodulatory biomaterials. These materials should be able to modulate the host immune response with the aim of creating a pro-regenerative microenvironment. In this review, we explore the potential of using of SPMs in the development of new immunomodulatory biomaterials and we propose insights for future research in this field.

## 1. The Acute Inflammatory Response to Biomaterials and Its Resolution

Implanted biomaterials will be recognized by the host immune system as a foreign body, triggering an immediate set of biological responses, leading ultimately to the development of an inflammatory and fibrotic reaction that is known as the Foreign Body Reaction (FBR) that presents a major challenge in the development and application of biomaterials and medical devices [1,2,3].

The FBR can be subdivided into different and sequential phases, starting with interactions between blood and the biomaterial leading to protein adsorption at the surface of the implant, followed by an acute inflammatory response that if persistent will lead to a chronic inflammation that will ultimately cause the formation of a fibrous capsule. We will herein focus on the onset of the inflammatory response, the acute inflammatory reaction as well as on its resolution.

The acute inflammatory reaction is of short duration and typically lasts from minutes to a few days. There are available in the literature extensive reviews on the different phases of the inflammatory response to biomaterials [1,4,5,6,7,8]. We will address general aspects of the acute response. The acute inflammatory response is mainly characterized by the formation of edema, through the exudation of fluid and proteins from plasma, together with the migration of leukocytes to the implant site [1]. The implantation of a biomaterial causes injury in the cells surrounding the implant site. This will lead to two different effects. Firstly and almost immediately, blood proteins adsorb to the biomaterial surface causing the activation of the coagulation cascade, the triggering of the complement system, and the activation and recruitment of inflammatory cells. Secondly, the injured cells will have an important role in the further recruitment of inflammatory cells through the release of DAMPs (Danger-associated molecular patterns), also known as danger signals or alarmins [8,9]. Polymorphonuclear leukocytes (PMNs) are the predominant inflammatory cell type in an acute response and the first ones to arrive at the implant site. PMNs will secrete ROS (Reactive oxygen species) and proteolytic enzymes that can corrode the surface of the biomaterial. Upon activation, PMNs also secrete interleukin (IL)-8 that will activate and recruit additional PMNs to the implant site. PMNs will also act as chemoattractants for monocytes and macrophages and for immature dendritic cells and lymphocytes through the production of the chemokines MCP-1 (Monocyte chemoattractant protein)-1 and MIP (Macrophage inflammatory protein)-1β. After two days, PMNs will disappear from the implant site, and the macrophage will be the dominant inflammatory cell [6,8,10]. At the implant site, macrophages can either polarize into M1 classically activated macrophages that will produce pro-inflammatory cytokines and release enzymes and ROS in an attempt to degrade the material or can polarize into M2 alternatively activated macrophages that will synthesize anti-inflammatory cytokines and growth factors that will facilitate tissue repair [11,12]. It is important to clarify that the M1/M2 macrophage phenotypic polarization is in fact a continuum of activation between these two phenotypes [12]. Additionally, M2 macrophages can be divided into different sub-groups that express different surface markers and have different associated functions: M2a, M2b, M2c, M2d, and M2_eff_. Among these sub-groups, M2a macrophages are the most associated with tissue repair and regeneration and interestingly M2_eff_ macrophages are induced by efferocytosis, a process that is prompted by SPMs as described in this review [13].

The termination of an inflammatory response was considered for many years to be due to the passive disappearance of the pro-inflammatory stimuli. It is now widely accepted that the resolution of an inflammatory response is an active and endogenous process that has the key role of preventing excessive and /or continued inflammation that would lead to a chronic state [14]. After an acute inflammatory response, the goal is to return to homeostasis in a complex process that involves a large number of molecules and cells of the immune system.

During an acute inflammatory response, cyclooxygenase-2 (COX-2) increases and leads to the production of pro-inflammatory mediators, such as leukotrienes (LTs) and prostaglandins (PGs) from arachidonic acid in phospholipidic membranes. Either these pro-inflammatory mediators can perpetuate the inflammatory response leading to a chronic state, or through the prostaglandin subtypes, PGE2 and PGD2 a change in the production of arachidonic acid-derived molecules is stimulated and anti-inflammatory mediators will be produced [15].

Therefore, the resolution of an inflammatory response can be explained in a simplistic way as a molecular switch of the lipid mediators from the production of pro-inflammatory mediators, such as LTs and PGs, to anti-inflammatory mediators originating from the same precursor. These anti-inflammatory mediators are collectively called specialized pro-resolving mediators (SPMs) [16].

SPMs are a group of lipidic molecules that have the ability to reduce inflammation through the activation of resolution pathways. In 1984 Serhan et al. [17] reported the isolation of the first SPM, lipoxin. Currently, this group of molecules, listed in Table 1, includes lipoxins (Lx), resolvins (Rv), protectins (PD), maresins (Mar), Cysteinyl-SPMs (Cys-SPMs) and n-3 docosapentaenoic acid (DPA)-derived SPMs (n-3 DPA-derived SPMs) [18]. SPMs are synthesized in the local inflammatory microenvironment at the onset of acute inflammation to compensate for the various pro-inflammatory mediators. Inflammation is a natural and important response of the host to injury and therefore SPMs will not block the process. When the immune system has performed its purpose SPMs will down-regulate the inflammatory response and ultimately lead to its resolution [19,20,21].

A generalized decrease of pro-inflammatory cytokines together with the phagocytosis of apoptotic PMNs and the removal of inflammatory debris are indicators of the resolution of the inflammatory response. SPMs will also facilitate tissue repair and regeneration through the decrease of pro-inflammatory mediators. When the resolution of an inflammatory response fails fibrosis and chronic inflammation will take place [22,23].

## 2. Mediators of the Resolution of the Inflammatory Response

**Figure 1:**
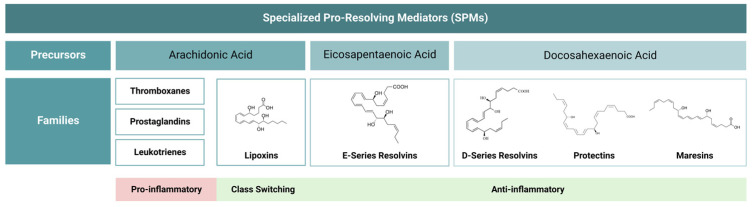
Specialized pro-resolving lipid mediators (SPMs) are produced from essential fatty acids. In the initiation of an inflammatory response, pro-inflammatory mediators such as prostaglandins and leukotrienes will induce the recruitment of inflammatory cells and the production of pro-inflammatory mediators. Afterward, a switch to the production of lipoxins will lead to the onset of the resolution of the inflammatory response.

Specialized pro-resolving mediators (SPMs) have a dual role since they have the ability to contain an acute inflammatory response and promote its resolution. SPMs are produced from dietary ω-3 polyunsaturated fatty acids (PUFAs) through the action of different enzymes. PUFAs are fundamental components of membrane phospholipids, and among other functions, they act as a precursor pool for lipid mediators since their main components include eicosapentaenoic acid (EPA) and docosahexaenoic acid (DHA) [24]. The different subtypes of SPMs are derived from arachidonic acid, EPA, DHA, and n-3 docosapentaenoic acid (DPA) as presented in Figure 1 [25,26,27].

### 2.1. Lipoxins (Lx)

The designation lipoxin is an acronym for “lipoxygenase interaction products”. There are two different Lx: LxA4 and LxB4. Lx can be synthesized by the lipoxygenation of arachidonic acid in epithelial cells, PMNs, and monocytes or through platelet/leukocyte interactions [19,28]. Lx is able to inhibit PMNs recruitment and activation by reducing vascular permeability; they also cause the non-phlogistic recruitment of monocytes, without degranulation or release of ROS, allowing the phagocytosis of apoptotic inflammatory cells as PMNs [29,30,31,32,33]. Interestingly, Lx also induces an increased release of transforming growth factor β1 (TGF-β1) by macrophages together with a decrease in IL-8 and MCP-1 [34]. Lx also has an effect on macrophage polarization from an M1 pro-inflammatory phenotype toward an M2 anti-inflammatory phenotype [31]. It has also been suggested that Lx can have anti-fibrotic properties [35].

### 2.2. Resolvins (Rv)

The label resolvins derive from “resolution-phase interaction products”. There are D-series resolvins (RvD1–RvD6) and E-series resolvins (RvE1–RvE4). Rv participates in different functions in the process of inflammation resolution as the regulation of the production of ROS and of different cytokines; the decrease of PMNs recruitment; promote the phagocytosis of apoptotic PMNs and promote debris clearance. Taken together, these effects will decrease the intensity of the response and facilitate tissue repair [29,36]. Additionally, it has been described that RvD1 promotes macrophage polarization to M2 anti-inflammatory phenotype [36,37] and that RvD4 decreases the release of neutrophil extracellular traps (NETs) [38].

### 2.3. Protectins (PD)

The name protectins were proposed for their general anti-inflammatory and protective actions. Protectin D1 (PD1) or neuroprotectin D1 (NPD1) and protectin X (PDX) were identified. In the case of PD1, if it is of neural origin, the designation NPD1 is used whereas the term PD1 is used to indicate its peripheral actions [39]. PD1/NPD1 decreases PMNs influx and strengthen the removal of apoptotic PMNs by macrophages [40]. NPD1 exhibits neuroprotective effects in the retina and in the central nervous system [41]. It has been reported that PD1 promotes M2 macrophage polarization and inhibits macrophage TNF (tumor necrosis factor)-α expression [42]. It is described that PDX has general anti-inflammatory and pro-resolving effects, and regulates inflammatory cell infiltration, namely PMNs [43]. Additionally, PDX causes a decrease in the expression of MIP-2 and MCP-1 in macrophages [44]

### 2.4. Maresins (Mar)

The term maresins come from “macrophage mediators in resolving inflammation”. Mar is produced by macrophages and has homeostatic functions. Mar1 and Mar2 have been identified. Maresins inhibit PMN influx and ROS production [45] and are powerful stimulators of macrophage efferocytosis leading to the clearance of apoptotic PMNs by macrophages [41,46]. Mar promotes the polarization of macrophages to an M2-phenotype [47]. Mar-1 activates leucine-rich repeat-containing G protein-coupled receptor 6 (LGR6), which is present in different tissues and promotes tissue repair and regeneration [48]. Additionally, Mar stimulated tissue regeneration in a planaria model [49].

### 2.5. Cysteinyl-SPMs (Cys-SPMs)

A new group of peptide-lipid conjugated SPMs was recently identified. This group includes three types of molecules: the resolvin conjugates in tissue regeneration (RCTRs); the protectin conjugates in tissue regeneration (PCTRs) and the maresin conjugates in tissue regeneration (MCTRs). It is reported that this group of SPMs presents anti-inflammatory and pro-resolving effects as well as regenerative properties [50]. It has also been reported its ability to stimulate tissue repair in a planaria model [51]. MCTRs have the ability to induce macrophage efferocytosis and to modulate macrophage polarization towards an M2 phenotype [25].

### 2.6. n-3 DPA-Derived SPMS

This new group of SPMs is derived from n-3 docosapentaenoic acid (DPA) and is generally designated as n-3 DPA-Derived SPMs. This group includes RvD_n-3DPA_, MaR_n-3DPA_, PD_n-3DPA_, and 13-series resolvins (RvTs) [39]. The n-3 DPA-derived SPMs also have important anti-inflammatory and pro-resolutive actions [27,52].

**Figure 2:**
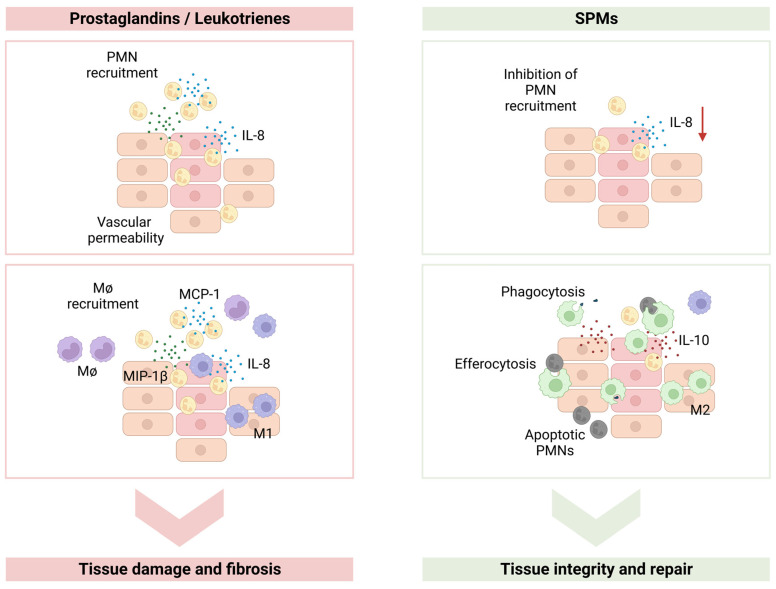
Specialized pro-resolving lipid mediators (SPMs) main actions. In an inflammatory response prostaglandins and leukotrienes will induce the recruitment of polymorphonuclear leukocytes (PMNs) that will produce high levels of IL (interleukin)-8 causing further recruitment of PMNs. PMNs will also produce MCP-1 (monocyte chemoattractant protein-1) and MIP (macrophage inflammatory protein)-1β causing the recruitment of monocytes/macrophages (Mφ), that will polarize into M1 pro-inflammatory macrophages, causing tissue damage. SPMs will cause a decrease in PMN recruitment, a decrease in the production of IL-8, and an increase in IL-10 secretion. SPMs will also lead to macrophage polarization towards an M2 anti-inflammatory phenotype and will induce macrophage efferocytosis. SPMs will cause the return to homeostasis and tissue repair.

Taking together the actions of the different SPMs described above, we can conclude that they exert rather interesting effects on the cells of the immune system (Figure 2). SPMs influence inflammatory cell recruitment and activation; regulate inflammatory cell apoptosis and have an effect on immune cell polarization. It is therefore of key importance to explore possible applications of these molecules in a therapeutic context to ensure an effective return to tissue homeostasis and to prevent fibrosis after an inflammatory response [24]. We will follow this review by exploring the potential of SPMs application in different medical conditions and in the field of biomaterial-based immunomodulation.

## 3. The Potential of SPMs in Medical Applications

Deficiencies in the resolution of inflammatory responses may lead to the development of chronic immune disorders. Furthermore, SPMs have an important potential in preventing and treating chronic inflammation and immune conditions [53]. The inflammatory response is in essence a protective reaction. Nevertheless, when exacerbated or when perpetuated in time can lead to tissue or organ damage or disease. The paradigm that inflammatory conditions occur due to the excessive production of pro-inflammatory mediators has changed. In fact, there is growing evidence that an inaccurate resolution of the inflammatory reaction, or an imbalance between pro-inflammatory mediators and SPMs are key factors in the emergence and advance of inflammatory conditions and several human diseases [54]. Therefore, the use of SPMs in the treatment of immune disorders appears as a rather promising approach.

Reports in the literature suggest that a deficient activity or production of SPMs is related to the pathogenesis of different inflammatory disorders. For example, an imbalance between pro-inflammatory mediators and SPMs was observed in inflammatory bowel disease [55]. In an evaluation of the lipidome of cerebrospinal fluid of Alzheimer’s disease patients, a decrease in the levels of SPMs was observed [56]. In the respiratory tracts of patients with chronic obstructive pulmonary disease or asthma, lower levels of LxA4 and PD1 were detected in comparison with healthy individuals [57]. It is also described that low serum levels of SPMs can be related to the development of hypertension [58]. Interestingly, it has also been suggested that SPMs are interesting candidates in the prevention of the cytokine storm in severe acute respiratory syndrome coronavirus 2 (SARS-CoV-2) patients [59].

Due to the growing evidence of the important role of SPMs, researchers have already started to explore the use of several different pro-resolving agents to treat inflammatory diseases. In an animal model of periodontal disease, the topical application of RvD1 in the tissues leads to an improvement in the condition of the disease causing a decrease in bone loss, and a decrease in the infiltration of PMNs [20,60]. In atherosclerosis, the process of macrophage efferocytosis is important to mitigate plaque progression. Since SPMs are known to induce macrophage efferocytosis, they appear as potential candidates to treat atherosclerotic plaques. In fact, RvD1, RvD2, and Mar1 were used in an animal model with established atherosclerosis and induced the decrease of several markers of plaque progression [61,62]. In an animal model of angiotensin-induced cardiac inflammation, the administration of RvD1 was demonstrated to have cardioprotective effects inhibiting cardiac remodeling and hypertension [63]. In rheumatoid arthritis, characterized by persistent joint inflammation, the administration of RvD1 was investigated using an animal model of this disease. The results obtained suggest that RvD1 led to a reduction of joint inflammation and consequently cartilage protection [64]. In an Alzheimer’s disease murine model, the administration of combined LxA4 and RvD1 leads to a decrease in inflammation [65]. Also, in an in vivo rat model of Parkinson’s disease, RvD2 caused a recovery of neuronal damage through the decreased expression of pro-inflammatory mediators [66,67]. It is currently accepted that inflammation is an important factor in the pathology of depressive disorders and that anti-inflammatory agents may have anti-depressive effects. It has been recently reviewed that intracranial infusions of different resolvins in rodent models of depression caused anti-depressive effects [68].

The use of SPMs is already being evaluated in clinical trials. The use of RvE1 is being assessed for topical application in ocular lesions [69]. The application of LxA4 analogs is under study for the treatment of infantile eczema and for the treatment of asthma [70,71].

This section clearly illustrates that the potential for SPMs application is vast. We have depicted some interesting examples to elucidate the possible range of applications.

## 4. SPMs in Biomaterial-Based Immunomodulation

The advent of tissue engineering and regenerative medicine led to the development of new biomaterials with the capability of enhancing tissue repair and regeneration. The recognition of the key importance of the immune system in tissue healing moved researchers to the development of a new class of biomaterials that present the ability to modulate inflammatory responses—the immunomodulatory biomaterials. Ideally, these biomaterials will control the host immune response to provide a favorable microenvironment able to promote tissue repair [72,73]. Immunomodulatory biomaterials have an interesting potential to modulate the FBR and therefore promote implant integration and function [74]. The majority of the immunomodulatory biomaterial-based strategies are focused on macrophages because they are highly plastic cells that can exhibit different phenotypes ranging from pro-inflammatory to anti-inflammatory [75,76]. Additionally, macrophages are important coordinators of tissue repair and regeneration [77].

Different strategies can be used to develop biomaterials with immunomodulatory properties such as (i) Changing the chemical properties of the material; (ii) tuning the physical characteristics of the biomaterial; (iii) incorporating bioactive molecules in the biomaterial either pro- or anti-inflammatory molecules or growth factors; (iv) combining biomaterials with cell therapies and (v) develop biomaterials derived from the extracellular matrix (ECM) of tissues or using ECM components [73,78,79,80,81].

**Figure 3:**
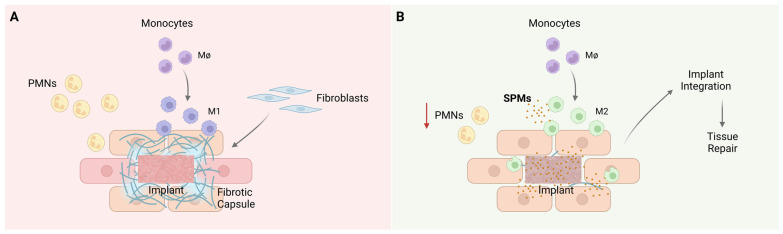
Foreign body response to biomaterials. (**A**) Implanted biomaterials will be considered a foreign body by the host immune system. Briefly, polymorphonuclear leukocytes (PMNs) will be the first immune cells to arrive at the implant site, followed by monocytes/macrophages that will polarize towards a pro-inflammatory M1 phenotype. Fibroblasts will be activated and will secrete collagen leading to the formation of a fibrous capsule around the implanted biomaterial. The implant will be isolated from the surrounding tissues and its function will be impaired. (**B**) An immunomodulatory biomaterial, for example, loaded with specialized pro-resolving lipid mediators (SPMs), will cause a decrease in inflammatory cell recruitment and will lead to macrophage polarization towards an M2 anti-inflammatory phenotype. This will enable implant integration as well as tissue repair.

Biomaterial-based immunomodulation through the use of SPMs is in our opinion a promising tool for the development of new and powerful immunomodulatory biomaterials. The acknowledgment of the active nature of the resolution of an inflammatory response, together with the understanding of the important effects of SPMs provides a new paradigm to address inflammatory conditions, improve implant integration and facilitate the return to tissue homeostasis [82], as represented in Figure 3. There are already some interesting and encouraging studies reported in the literature that combine the application of different types of SPMs with biomaterials, which we describe in the following paragraphs and are summarized in Table 2.

Regarding the use of lipoxins, Wang et al. [83] have developed a polyisocyanopeptide hydrogel loaded with the antimicrobial agent doxycycline and LxA4 for periodontal treatment. When applied in a dog model, this hydrogel leads to the decrease of sub-gingival bacterial loading and to the decrease in the levels of the pro-inflammatory cytokine IL-8. Reis et al. [84] produced poly-lactic-co-glycolic acid (PLGA) microparticles loaded with LxA4 for the treatment of skin ulcers. When tested in a rat model of skin lesion the microparticles caused a reduction of inflammatory cell numbers, promoted the closure of the wound, reduced the levels of IL-1β and TNF-α, and increased the levels of TGF-β. Sun et al. [85] developed calcium carbonate nanoparticles loaded with a LxA4 agonist for the treatment of endometriosis. They observed an enhancement in macrophage efferocytosis as well as a decrease in the levels of pro-inflammatory cytokines.

We have used LxA4 and RvD1 to dampen the inflammatory response to implanted 3D chitosan (Ch) scaffolds [86]. After implanting the Ch scaffolds in a rodent air-pouch model, we performed local administration of either LxA4 or RvD1 every 12 h for 3 days. We were able to observe a shift in the macrophage polarization profile towards M2, as well as a general decrease in the levels of pro-inflammatory cytokines. Afterward, we incorporated RvD1 in the Ch scaffolds [87]. This biomaterial was evaluated in a rodent air-pouch model and caused a decrease in the recruitment of inflammatory cells together with higher numbers of M2 macrophages and lower numbers of M1 macrophages and a general decrease in the levels of pro-inflammatory cytokines. We have also tested the scaffolds in a rat femoral critical size defect model and concluded that RvD1 contributed to the formation of new bone with improved trabecular thickness [88]. Some other authors have explored the use of resolvins in biomaterial-based immunomodulation. Sok et al. [89] developed a poly(ethylene) glycol (PEG)-based hydrogel to release aspirin-triggered (AT) RvD1 and IL-10 in a murine model of dorsal skinfold window chamber. They observed an enhancement in the recruitment of anti-inflammatory myeloid cells with the combined delivery of AT-RvD1 and IL-10. In another study using the same in vivo model [90], the authors tested PLGA scaffolds loaded with AT-RvD1 and reported a decrease in the recruitment of PMN leukocytes together with an increase of M2 macrophages. Shi et al. [91] developed a polycaprolactone vascular graft loaded with AT-RvD1, and in an in vivo model where they performed the replacement of the rat abdominal aorta by a vascular graft, concluded that the presence of AT-RvD1 in the graft decreased PMN recruitment and activation and caused an increase in the number of M2 macrophages. Yin et al. [92] with the aim of improving bone tissue repair through the control of inflammation during the healing process developed a gold nanocage loaded with RvD1 to induce M2 macrophage polarization. They were able to observe an increase in macrophage polarization towards M2 phenotype both in vitro using a macrophage cell line and in vivo in a mouse femoral defect model. Lu et al. [93] designed a catechol-chitosan hydrogel incorporated with acetalized cyclodextrin nanoparticles loaded with RvE1 for the treatment of chronic wounds. When tested in vitro with a macrophage cell line an increase in the production of the anti-inflammatory cytokine IL-10 was observed. In a rat wound healing model, the hydrogel stimulated the closure of the wound. Jiang et al. [94] investigated the effect of RvD1 in bone regeneration using a rat calvarial defect model. They produced collagen scaffolds impregnated with a pluronic hydrogel with and without the incorporation of RvD1. The results obtained suggest that RvD1 improved bone formation and vascularization. The authors suggest that this effect was due to the ability of RvD1 to control the inflammatory microenvironment although they did not study the effects on immune cells or on immune mediators.

As to the use of maresins, Miranda et al. [95] produced polylactic acid nanoparticles incorporated with Mar2 for intestinal mucosal wound repair. They observed in a biopsy-wounding model that the administration of the nanoparticles promoted mucosal repair and reduced the number of infiltrating immune cells.

We are certain that this is just the first group of studies combining the use of biomaterials and SPMs. The promising and interesting results yielded in these reports will cause increased attention in this area of research. Additionally, there are yet several subtypes of SPMs with powerful anti-inflammatory and pro-resolutive actions that remain to be explored.

SPMs are challenging to work with because they are unstable molecules and have a diminutive biological half-life that was observed both in in vitro and in in vivo studies. One possibility to overcome this limitation is the development of more stable analogs. It is reported in the literature for example the development of an oxidation-resistant metabolic stable analog of the n-3 DPA-derived protectin D1 [96]; the production of lipoxin A4 stable analogs [97]; and also of analogs for resolvin D1 [98]. Alternatively, in a previous work of ours with chitosan scaffolds incorporated with RvD1 [87] we used lyophilization, which resulted in higher RvD1 activity since this procedure is a lipid stabilizer and is reported to increase the stability of different pharmaceutical formulations. In a different study conducted by de Prinse et al. [99], another solution was explored, SPMs-loaded micelles were developed to address the challenge of the long-term use of these molecules. These micelles were revealed to be non-cytotoxic and their potential use in vivo must now be explored.

## 5. Conclusions and Future Perspectives

SPMs have an essential role in the resolution of an inflammatory acute reaction and in the return to homeostasis. SPMs have important modulatory effects in different cells of the immune system, and although their actions are broad, it is possible to summarize in three key functions the main effects of this family of pro-resolutive molecules:

(i). SPMs can inhibit the recruitment and activation of immune cells. These molecules have an important role for example in the recruitment of PMNs to the inflammatory site and can decrease the production of pro-inflammatory cytokines by inflammatory cells.
(ii). SPMs have an important effect on the modulation of the polarization of immune cells. For example, these molecules induce the polarization of macrophages towards an M2 anti-inflammatory phenotype.
(iii). SPMs regulate macrophage phagocytic capacity, leading to increased elimination of apoptotic immune cells by macrophages in a process called efferocytosis.

Taking into consideration these important effects, we believe that SPMs represent a rather promising strategy to stop an inflammatory response and mitigate the potential undesired side effects such as tissue damage or fibrosis. Moreover, SPMs seem to be advantageous in comparison with other anti-inflammatory or immunosuppressive molecules because they will promote the resolution of an inflammatory reaction without the unwanted immunosuppressive side effect. Additionally, SPMs will not suppress the positive effects of an inflammatory reaction in terms of host response against pathogens and in the promotion of tissue repair and return to homeostasis.

The potential action of SPMs must be thoroughly evaluated. In our opinion, research efforts should be made to clarify several different aspects. In the following part of the manuscript, we list some important research lines that are in our opinion worth exploring:

1. Extend the studies already carried out to all SPMs and address possible new applications: SPMs represent a large family of lipidic molecules, and many of them were not tested in biomaterial-based applications. In Section 4, where we performed a review of the SPMs used for biomaterial-based immunomodulation we were only able to find research works using LxA4, RvD1, RvE1, and Mar2. Many other SPMs still need to be explored.
2. Explore the potential synergic effect of SPMs: The combination of more than one SPM within a biomaterial could have interesting synergic effects. In Section 3, we discuss the use of combined LxA4 and RvD1 in an animal model of Alzheimer’s disease that caused a significant decrease in the inflammatory response. Therefore, the combination of different SPMs should be addressed.
3. Investigate the potential of protectins: The application of protectins in the development of new functional biomaterials is clearly underexplored. More specifically, neuroprotectins that have been identified as having neuroprotective effects could represent an advance in the development of biomaterials for neuroscience applications.
4. Understand the regenerative capacity of the cysteinyl-SPMs: The recently identified subclass of SPMs, the cysteinyl-SPMs, includes the resolvin, protectin, and maresin conjugates in tissue regeneration. It is reported that this group of molecules besides having pro-resolutive actions have regenerative properties. Since we are seeking ideal biomaterials for tissue engineering and regenerative medicine, these molecules appear as ideal candidates for the development of new biomaterials with immunomodulatory properties.

A comprehensive understanding of the biological actions of SPMs will provide key information for the regulation of inflammation, its resolution, and the return to homeostasis. We are certain that the application of SPMs in the development of new biomaterials will be a milestone in the field of immuno-engineering.

## Figures and Tables

**Table 1:** Specialized pro-resolving mediators (SPMs) family.

SPM(s)	Subtypes	Precursor
Lipoxins	LxA4 and LxB4	Arachidonic acid
Resolvins	E-series Resolvins (RvE1–RvE4) D-series Resolvins (RvD1–RvD6)	Eicosapentaenoic acid Docosahexaenoic acid
Protectins	Protectin D1/Neuroprotectin D1 PDX	Docosahexaenoic acid
Maresins	Mar1 and Mar2	Docosahexaenoic acid
Cysteinyl-SPMs	MCTR1–MCTR3 PCTR1–PCTR3 RCTR1–RCTR3	Docosahexaenoic acid
n-3 DPA-derived SPMs	(RvD1, RvD2, RvD5)_n-3DPA_ (PD1, PD2)_n-3DPA_ (Mar1–Mar3)_n-3DPA_ RvT1–RvT4	n-3 Docosapentaenoic acid

**Table 2:** SPMs are used in biomaterial-based immunomodulation.

Specialized Pro-Resolving Mediators (SPMs)	Biomaterial	Immunomodulatory Outcome	Reference
LxA4	Polyisocyanopeptide hydrogel	Decreased levels of IL-8	[82]
LxA4	Poly-lactic-co-glycolic acid microparticles	Reduced numbers of inflammatory cells Reduced levels of IL-1β and TNF-α Increased levels of TGF-β	[83]
LxA4 agonist	Calcium carbonate nanoparticles	Enhanced macrophages efferocytosis Decreased levels of pro-inflammatory cytokines	[84]
RvD1	Chitosan scaffolds	Higher numbers of M2 macrophages Lower numbers of M1 macrophages Decrease in pro-inflammatory cytokines	[86]
RvD1	Poly(ethylene) glycol-based hydrogel	Decreased recruitment of PMN leukocytes Increased numbers of M2 macrophages	[88]
RvD1	Polycaprolactone vascular graft	Decreased PMN recruitment Increased numbers of M2 macrophages	[89]
RvD1	Gold nanocage	Increased numbers of M2 macrophages	[91]
RvE1	Catechol-chitosan hydrogel	Increased production of IL-10	[92]
Mar2	Polylactic acid nanoparticles	Reduced numbers of infiltrating immune cells	[94]

## Data Availability

Not applicable.

## Abbreviations

FBR: Foreign Body Reaction; PMNs: Polymorphonuclear leukocytes; DAMPs: Danger-associated molecular patterns; ROS: Reactive oxygen species; IL: interleukin; MCP: Monocyte chemoattractant protein; MIP: Macrophage inflammatory protein; COX-2: cyclooxygenase-2; LTs: Leukotrienes; PGs: Prostaglandins; SPMs: Specialized pro-resolving mediators; Lx: Lipoxins; Rv: Resolvins; PD: Protectins; Mar: Maresins; Cys-SPMs: Cysteinyl- Specialized pro-resolving mediators; DPA: Docosapentaenoic acid; PUFAs: Polyunsaturated fatty acids; EPA: Eicosapentaenoic acid; DHA: Docosahexaenoic acid; TGF: transforming growth factor; NETs: Neutrophil extracellular traps; NPD: Neuroprotectin; TNF: tumor necrosis factor; RCTRs: Resolvin conjugates in tissue regeneration; PCTRs: Protectin conjugates in tissue regeneration; MCTRs: Maresin conjugates in tissue regeneration; SARS-CoV 2: Severe acute respiratory syndrome coronavirus 2; PLGA: Poly-lactic-co-glycolic acid; Ch: Chitosan; PEG: Poly(ethylene) glycol; AT: Aspirin-triggered.
